# Monitoring Crop Growth During the Period of the Rapid Spread of COVID-19 in China by Remote Sensing

**DOI:** 10.1109/JSTARS.2020.3029434

**Published:** 2020-10-07

**Authors:** Yan Wang, Dailiang Peng, Le Yu, Yaqiong Zhang, Jie Yin, Leilei Zhou, Shijun Zheng, Fumin Wang, Cunjun Li

**Affiliations:** 1 Key Laboratory of Digital Earth Science, Aerospace Information Research InstituteChinese Academy of Sciences 12381 Beijing 100094 China; 2 Department of Earth System ScienceTsinghua University 12442 Beijing 100084 China; 3 Center for Satellite Application on Ecology and EnvironmentMinistry of Ecology and Environment 251406 Beijing 100006 China; 4 School of Surveying and Land Information EngineeringHenan Polytechnic University 12561 Jiaozuo 454003 China; 5 Institute of Remote Sensing and Information Technology ApplicationZhejiang University 12377 Hangzhou 310058 China; 6 Beijing Research Center for Information Technology in Agriculture 205339 Beijing 100097 China

**Keywords:** Coronavirus disease 2019 (COVID-19), crop growth, remote sensing

## Abstract

The status of crop growth under the influence of COVID-19 is an important information for evaluating the current food security in China. This article used the cloud computing platform of Google Earth Engine, to access and analyze Sentinel-2, MODIS, and other multisource remote sensing data in the last five years to monitor the growth of crops in China, especially in Hubei province, during the period of the rapid spread of COVID-19 (i.e., from late January to mid-March 2020), and compared with the growth over the same period under similar climate conditions in the past four years. We further analyzed the indirect effects of COVID-19 on crop growth. The results showed that: the area of the crops with better growth (51%) was much more than that with worse growth (22%); the crops with better and worse growth were mainly distributed in the North China Plain (the main planting areas of winter wheat in China) and the South China regions (such as Guangxi, Guangdong province), respectively. The area of the crops with a similar growth occupied 27%. In Hubei province, the area of the crops with better growth (61%) was also more than that with worse growth (27%). It was found that there was no obvious effect from COVID-19 on the overall growth of crops in China during the period from late January to mid-March 2020 and the growth of crops was much better than that during the same period in previous years. The findings in this study are helpful in evaluating the impact of the COVID-19 on China's agriculture, which are conducive to serve the relevant agricultural policy formulation and to ensure food security.

## I. Introduction

The coronavirus disease 2019 (COVID-19) pandemic has led to worldwide human health issues [1]. On 23 January 2020, Wuhan, Hubei Province, China, announced the blockade of the traffic to prevent the spread of the COVID-19. Since then, the measures of quarantines and other restrictions have been implemented in China [2]–[4]. Until 29 January 2020, 31 provinces in China have activated the first-level public health emergency response, and the confirmed cases have been found in nearly 300 cities (districts) in China according to the statistical data of COVID-19 from the National Health Commission (NHC) of the People's Republic of China. The increasing rate of the confirmed cases has declined since 19 February 2020. On 11 March 2020, the statistical data released by NHC indicated that China had made significant progress in the prevention and control of the COVID-19 pandemic [5]. During this time, the necessary measures of limiting human movement are inevitably causing economic shocks [1], especially for the tertiary industry in China during the period of COVID-19 outbreak. The tourism, entertainment, and transportation industries have all suffered varying degrees of losses [6]–[8]. The COVID-19 pandemic also affects agricultural activities and food systems worldwide. The immediate impacts include food security, labor availability, farm system resilience, agricultural system connectivity, and other impacts and questions [1], [9]–[11]. In China, a series of policies have been issued to reduce the impact of COVID-19 pandemic. For agriculture, many corresponding measures have also been made, such as spring field management and tillage preparation are required to be taken as critical agricultural tasks. China is one of the largest agricultural countries in the world and characterizes large population, planting area, and crop yield. The status of crop growth in China under the influence of COVID-19 is an important information for governments to make the related policies timely for food security worldwide.

The North China Plain is the main grain production area in China. The main food crops are winter wheat and summer maize, and the main farming system is the winter wheat-summer maize rotation system. Most areas of the North China Plain have a typical temperate monsoon climate, with relatively little precipitation, mostly concentrated in summer and heavy rains. The precipitation area is unevenly distributed, with little rain in winter and spring, and severe drought in spring. Due to the long-term implementation of single no-tillage and straw returning to the field, and the compaction of the soil by agricultural mechanized harvesting, the soil degradation characteristics of effective tillage are obvious [12]–[16]. The southern region is dominated by the tropical and subtropical monsoon climate, with mild winters and less rain, and the climate is relatively humid. The windward slope of mountain farmland has more precipitation. The eastern coastal areas are greatly affected by typhoons in summer and autumn. The main crops in winter are rapeseed, followed by flax [17], [18]. The main winter crops in China are winter wheat, cabbage, and rape. Climate changes have a certain impact on the growth and development of crops, planting zoning, planting area, and yield, etc. The growth period of each crop shows different characteristics according to the suitability of temperature, precipitation, light, and other climatic factors [19].

Food security is a broad subject that includes crop growth, crop yield, diseases, field management, international trade, etc. [20], [21]. The status of crop growth is impacted by water stress, pest stress, field management (e.g., irrigation, fertilization), and also finally affects the crop yield [20]–[22]. *In situ* observations for the status of crop growth in a small plot or region are generally accurate but their spatial coverage is often inadequate [20]. Large area or national scale *in situ* observations for crop growth are often limited by time-consuming and different standards or methods [20]–[22]. A remote sensing method creates an opportunity for solving the above-mentioned problem because the remote sensing data provided large spatial coverage and never stop acquiring images for the earth's surface [23], [24]. Therefore, crop monitoring by remote sensing has been widely used by agricultural departments worldwide and scientific institutions [20]–[22], [25], [26]. Crop growth monitoring by remote sensing characterizes multiple spatial and temporal scales [20]–[22]. Many remote sensing data have been used for this monitoring, such as low spatial resolution and high temporal resolution images from National Oceanic Atmospheric Administration advanced very high-resolution radiometer, Fengyun 3, Satellite Pour l'Observation de la Terre vegetation, and moderate-resolution imaging spectroradiometer (MODIS), and these images are generally used for large regions. The images, such as Landsat and Sentinel with the medium or high spatial resolution and low temporal resolution, are more advantaged generally when monitoring crop growth for the local region because of their medium or high spatial resolution, while it would cost too much time when applying them on a wider scale, such as national and global scale [20]–[22]. Crops growth monitoring in remote sensing includes monitoring the growth of cereal crop seedlings, growth status, and changes, etc. [23], [24], [27]. Many methods or models are developed for crop growth monitoring by remote sensing and the vegetation index derived from those remote sensing data is widely used [28]–[30], such as normalized difference vegetation index, enhanced vegetation index (EVI), and so on [21], [22], [31], [32] because the vegetation indices can reflect crops seasonal dynamic [29], [33], [34]. By comparing those vegetation indices among different years, we could find out the current status of crop growth under similar meteorological conditions, which is one of the major factors to affect the crop growth and the values of vegetation indices [20]–[22], [35]–[39].

The traditional remote sensing methods, however, limited by the large volume of data and inefficient local computing, limit the real-time monitoring of crops. For example, we have to cost much time in downloading necessary data from different website and integrate them in various ways if we propose to clarify a phenomenon. However, google earth engine (GEE) provides the opportunity for resolving such a problem. Relying on google's cloud infrastructure, GEE stores petabytes and it also provides rich application program interfaces and interactive development environment. This makes it possible to efficiently deal with these data and use them to monitor crops synchronously [40]. At present, agricultural applications based on GEE mainly include crop identification and crop monitoring. Crop identification mainly includes crop classification and crop mapping. GEE built-in algorithms, such as random forest, support vector machines, and classification and regression trees [41]–[43] as well as some classification methods based on the phenological characteristics of different crops, perform well when applied in crop classification [44]–[46]. Crop monitoring mainly includes real-time growth monitoring and yield prediction [47], [48]. For example, by comparing vegetation index anomalies and rainfall anomalies in a certain period of several years, the potential agricultural risk areas are extracted and expert decision making is used to achieve the purpose of agricultural early warning. Moreover, a crop growth model that combines vegetation index and meteorological factors also successfully predict the yield of crops [48]. In addition, GEE is also applied to the serviceability assessment of agricultural land or the irrigation dynamics of irrigated agriculture [49], [50]. For example, Jillian *et al*. combined 18 years of Landsat data (including Landsat 5, 7, and 8) and weather data, etc., based on GEE's built-in random forest, and classification and decision tree methods to plot the year-by-year irrigation dynamics in the Republican River Basin, which provides effective support for the policy formulation of irrigated agriculture.

In this study, the period from January 23 to March 11, 2020 was regarded as the rapid spread period of the COVID-19 in China. Multisource remote sensing data and meteorological data were selected to monitor the growth of the crops throughout China, particularly in Hubei province.

## II. Data and Method

### A. Remote Sensing and Meteorological Data

The land use/cover data in China are mainly obtained from the latest land use and land cover (LUCC) product in 2015, which has a spatial resolution of 30 m, and produced by the Institute of Geographic Sciences and Natural Resources Research, Chinese Academy of Sciences. This LUCC product was produced by the human interpretation method based on Landsat 8 operational land imager images [51]–[53]. The first-level classification of this LUCC product includes cultivated land, forest land, grassland, etc. Then a second-level classification was carried out based on the first-level classification, such as the cultivated land was divided into the paddy field and dry land. The overall accuracy of the remote sensing interpretation of paddy field and dry land is about 84% and 93%, respectively [51]–[53]. The dry land was extracted as the study area based on the results of the first-level classification. In addition, International Geosphere–Biosphere Programme (IGBP) land cover classification of MCD12Q1 (463 m) from 2015 to the latest (i.e., 2018) product was selected and the pixels that are cropland in four years were extracted by using GEE.

The reflectances during January 23 to March 11, from 2016 to 2020 were extracted from MOD09GA and Sentinel-2 multispectral satellite (Sentinel-2 MSI), and acquired from the GEE data catalog. The former has a spatial resolution of 463 m and a temporal resolution of one day [54]. The latter was provided by the European Space Agency, and it has a spatial resolution of 10 m and a temporal resolution of ten days [55]. The reflectance was used to calculate the indicators and to monitor the crop growth by the cloud computing platform of GEE.

We obtained the daily temperature and precipitation data of medium-range weather forecasts (ECMWF) during January 23 to March 11, from 2016 to 2019 through GEE [56]. The data have a spatial resolution of 0.25°. The climate data are used for extracting the year with the most analogous climate to 2020.

### B. Finding the Climate Year Most Analogous to 2020

We intended to compare the vegetation indices difference between the year 2020 and the year with the most analogous climate to 2020. The method developed by the CGIAR Research Program on Climate Change, Agriculture and Food Security (hereafter called CCAFS) was adopted in this study [57]. CCAFS attempts to find a future scenario, which is similar to the current climate by using a series of climate data. CCAFS first selects a region as the target and collects climate data (such as temperature and precipitation record each month in a past year) of the target region. Then a combination of the target region climate data and future grid climate data is used to calculate the climate similarity between the world and the target area.

Here, we tailored the CCAFS method to fit our goal of finding the year with the most analogous climate compared with the climate condition during the rapid spread period of the COVID-19 in China in 2020. The similarity of climate conditions between twom years becomes higher when the value of CCAFS becomes smaller. The expression of the method is as follows:

\begin{equation*}
{\rm{CCAFS}} = {\left({\mathop \sum \limits_{i = 1}^m \mathop \sum \limits_{j = 1}^n {W_{ij}}*{{\left({{V_{ij2020}} - {V_{ijp}}} \right)}^2}} \right)^{1/2}}\tag{1}
\end{equation*}

where the climate in 2020 and past years is described as a vector of *m* sequential daily mean values for *n* variables. Here the value of *m* is 48, which represents days from 23rd January to 11th March. The value of *n*, which is 2, represents the temperature and precipitation, respectively. *V_ij_*_2020_ and *V_ijp_* denote the value of climate variable *j* at time *i* in 2020 and the value of climate variable *j* at time *i* in one of the past years between 2016 and 2019, respectively. Here *W_ij_* refers to the weight of *V_ij_*. The temperature in winter is the more major climate factor than the precipitation that influences the crop growth of winter crop. For the areas in the northeast and northwest China, the weather in the winter season characterizes cold and low precipitation, and causes few crops planted in most of these regions, while for most of the area in the south of China, due to sufficient rainfall, the growth of crops in winter is mainly controlled by the temperature. Therefore, the weight of temperature is assigned 0.7 and the weight of precipitation is assigned 0.3 [57]. Based on the above-mentioned method, we calculated the similarity degree with the climate of 2020 for each year from 2016 to 2019 and extracted the year with minimal CCAFS as the climate analogous year for each pixel.

### C. Crop Growth Monitoring by Remote Sensing Data

In this study, EVI was selected as the indicator for monitoring crop growth because it was developed to maintain high sensitivity in dense vegetation areas and reduce the influence of soil and atmosphere [58], [59]. Based on the cloud computing platform of GEE, we collect the reflectance data of MODIS (MOD09GA) and Sentinel-2. We first remove pixels labeled cloud and filled these gaps with the weighted average method by using their previous and subsequential images. Then the average EVI during the period January 23 to March 11 from 2016 to 2020 was calculated using the gap-filled reflectance data from MOD09GA in the whole of China and from Sentinel-2 in Hubei province, respectively. We then masked the areas with EVI less than 0.1 because these areas are convinced to be areas of barren rock, sand, or snow [60], [61]

\begin{equation*}
{\rm{EVI}} = 2.5 \times \frac{{{\rho _{{\rm{NIR}}}} - {\rho _{{\rm{RED}}}}}}{{{\rho _{{\rm{NIR}}}} + 6.0 \times {\rho _{{\rm{RED}}}} - 7.5 \times {\rho _{{\rm{BLUE}}}} + 1}}\tag{2}
\end{equation*}

where ${\rho _{{\rm{NIR}}}}$, ${\rho _{{\rm{RED}}}},$ and ${\rho _{{\rm{BLUE}}}}$ are the reflectance in near-infrared, red, and blue wavelengths, respectively. These three bands are 703.9 nm in Sentinel-2A, 703.8 nm in Sentinel-2B, and 841-876 nm in MOD09GA; 664.5 nm in Sentinel-2A, 665 nm in Sentinel-2B, and 620–670 nm in MOD09GA; 496.6 nm in Sentinel-2A, 492.1 nm in Sentinel-2B, and 459–479 nm in MOD09GA.

The year with the most analogous climate condition in the past four years was found per pixel based on the CCAFS method, and the average EVI in this year was used to compare with the average EVI in 2020, to evaluate the growth of crops during the rapid spread period of the COVID-19 in China. The relative change rate (CR) of EVI between them was calculated by the following equation on the cloud computing platform of GEE:

\begin{equation*}
{\rm{CR}} = \frac{{{\rm{EV}}{{\rm{I}}_{{2020}}} - {\rm{EV}}{{\rm{I}}_{{\rm{similar}}}}}}{{{\rm{EV}}{{\rm{I}}_{{\rm{similar}}}}}} \times 100\%\tag{3}
\end{equation*}

where EVI_2020_ and EVI_similar_ represent the average EVI in 2020 and in the year with the closest LST compared with the LST in 2020, respectively.

We assumed that the crop growth was better, similar, or worse in 2020 compared with that in the past four years when CR > 5%, −5% ≤ CR ≤ 5%, or CR < −5%, respectively, and calculated the proportion of area (PA) for the crops with better growth, similar growth, and worse growth at the provincial scale in China and city-scale in Hubei province, respectively

\begin{equation*}
{\rm{PA}} = \frac{{{A_i}}}{{{A_T}}} \times 100\%\tag{4}
\end{equation*}

where ${A_i}$ represent the area of the crops with better growth, similar growth, or worse growth, and ${A_T}$ represent the total area of crops.

Given that the two kinds of land cover data remain some uncertainties. For example, agriculture is sometimes under-represented and labeled as natural vegetation in some tropical areas in MCD12Q1 IGBP classification data because the cropland field sizes may be too small to be identified. The overall accuracy of the paddy field and dry land in Landsat land cover map is about 84% and 93%, respectively. Such uncertainties might influence our results. We, therefore, investigated the difference in crop growth based on different land cover products (i.e., LUCC and MCD12Q1) to evaluate the consistency of results based on the two land cover data. In addition, the crop growth based on MODIS EVI and Sentinel-2 EVI in Hubei province was compared, to find out the influence of the crop growth monitoring because of different spatial resolutions.

To check the indirect influence of COVID-19 pandemic on crop growth during the period of its rapid spread in China, the number of the city-scale cumulative confirmed cases released by the NHC on March 11 was used, and the correlation between the crop growth and the cumulative confirmed cases of COVID-19 was examined at the city scale. Because the cumulative confirmed cases in Wuhan is much higher than that in other cities, our correlation analysis excluded Wuhan city.

## III. Results and Discussion

### A. Spatial Distributions of the Crop Growth at Different Scales

**Fig. 1.**
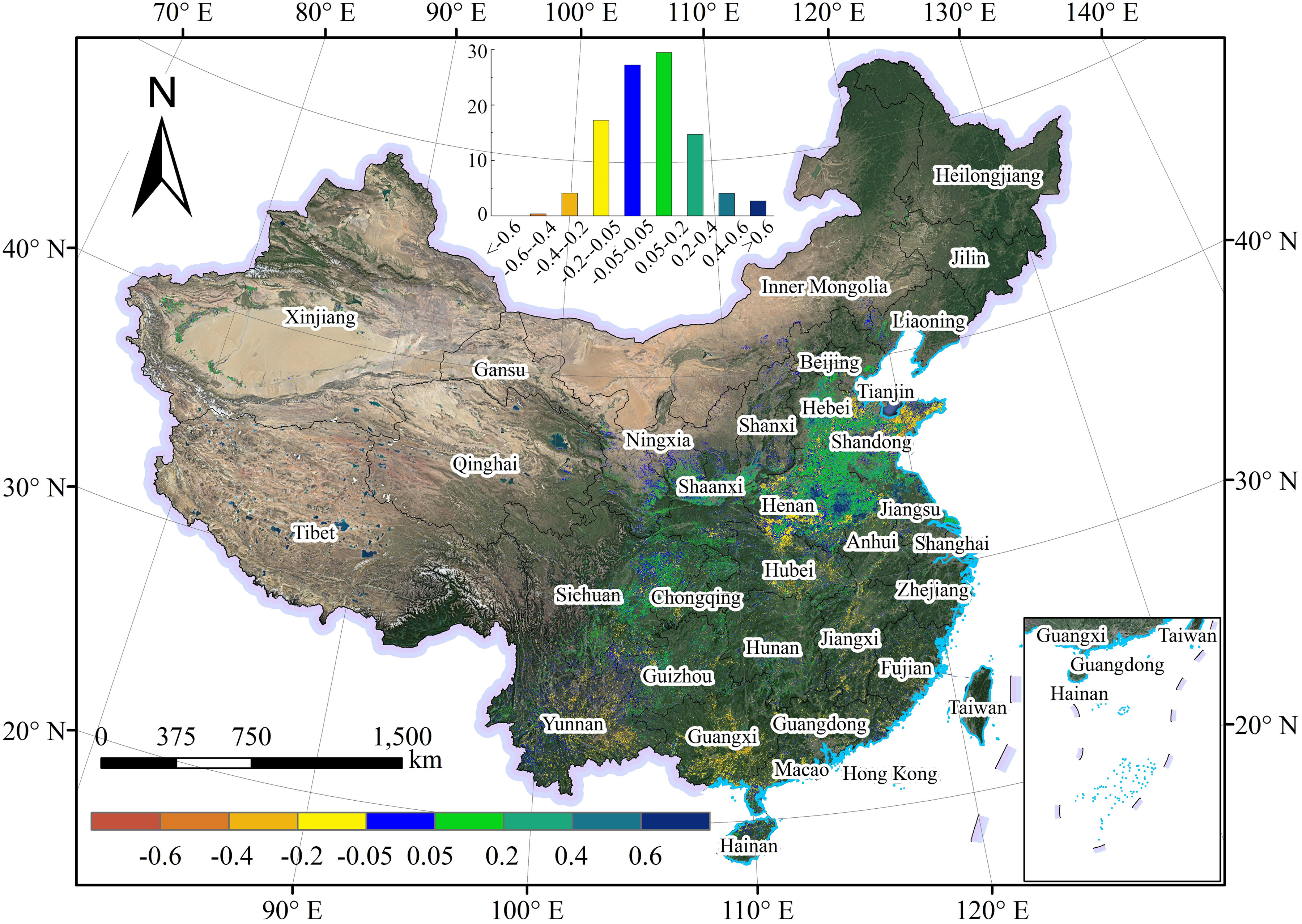
During the period of the rapid spread of COVID-19 in China, the distributions of CR of crops on google earth image compared to that in the past four years under similar meteorological conditions in China.

During the rapid spread period of COVID-19 in China, the crop growth monitoring at the pixel scale was displayed in Fig. 1, which was based on MOD09GA EVI. In Fig. 1, crops with worse growth, similar growth, or better growth were distributed in the interval of CR < −5%, −5% ≤ CR ≤ 5%, and CR> 5%, respectively. It was found that the winter crops were mainly distributed in the south of 40 °N and the east of 100 °E. The regions in the North and Northwest China, such as the provinces of Heilongjiang, Jilin, Inner Mongolia, Xinjiang, and Qinghai, and Tibet have few winter crops. The statistics of the crop growth in these provinces and some other provinces or regions with the small area of crops (e.g., Hong Kong, Macau, Taiwan, and so on) were not displayed in Fig. 3, which presented the PA for the crops with better growth, similar growth, and worse growth at the provincial scale in China and city scale in Hubei province, respectively.

**Fig. 2.**
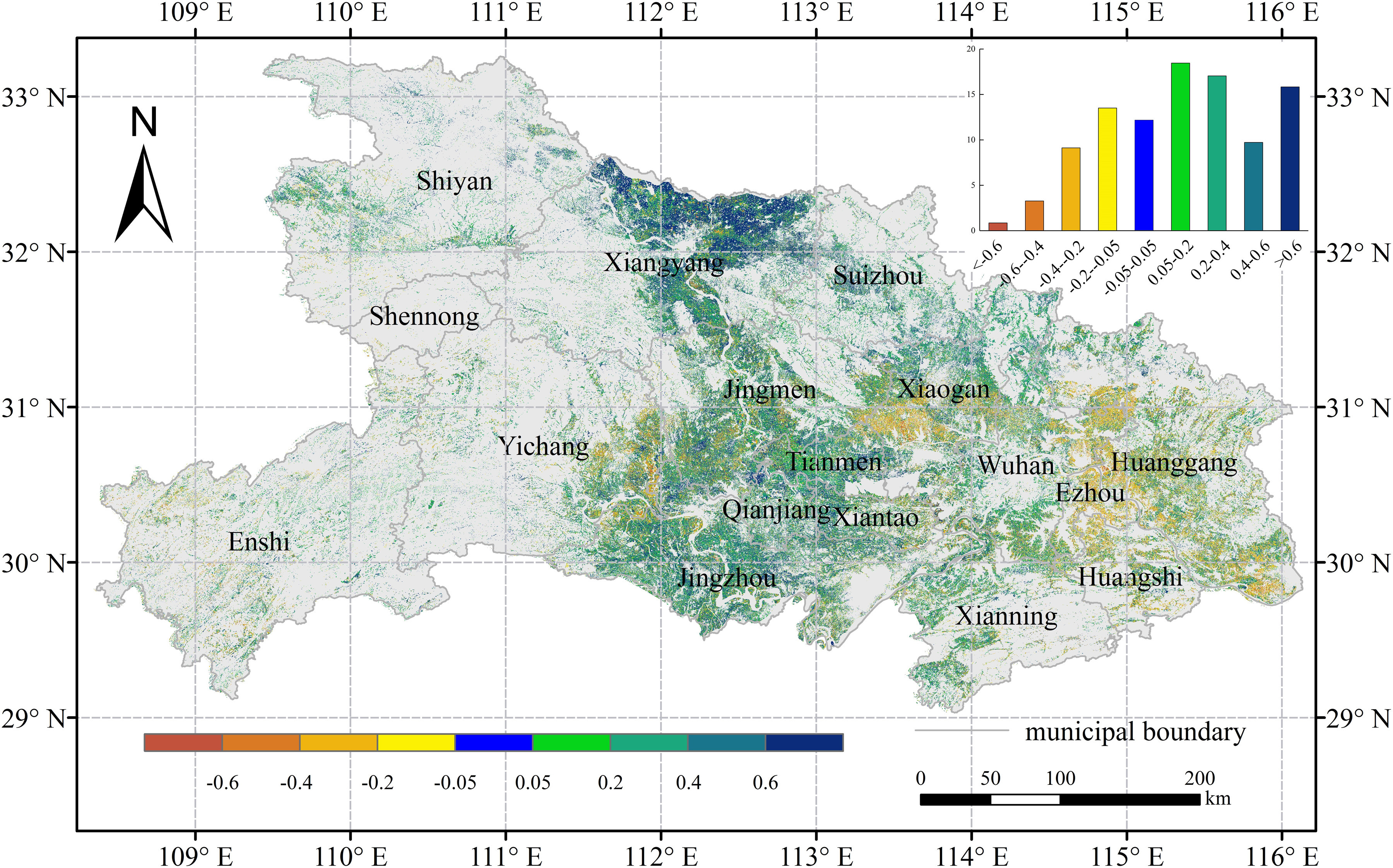
During the period of the rapid spread of COVID-19 in China, the distributions of CR of crops compared with those in the past four years under similar meteorological conditions in Hubei province.

The CR mainly concentrated on the interval of −0.2–0.4, which accounts for almost 90% of all pixels (see Fig. 1). Compared with the crop growth in the past four years under similar meteorological conditions, the crop growth was generally much better during the rapid spread period of COVID-19 in China in 2020. The PA for the crops with better growth, similar growth, and worse growth were 51%, 27%, and 22%, respectively. The relative CR for better growth and worse growth was 17% and 14%, respectively (see Fig. 1), and that for a similar growth was approximately 0%. Spatially, the region with better crop growth has covered most of the winter crop planting area and was mainly located in the North China Plain. The crops with the best growth occurred in the east of Henan and the CR is more than 60% (see Fig. 1). The regions with worse crop growth were mainly located in the southern China and eastern Shandong Province and southern Henan Province. The crop with a similar growth was mainly distributed in southern China (such as Yunnan and Guizhou provinces) and the provinces of Ningxia and Shaanxi. The crop growth based on the EVI derived from sentinel-2 reflectance data showed a consistent spatial distribution with the results from MOD09GA EVI in Hubei province (see Fig. 2). In Hubei province, the CR of crops greater than -0.4 occupies about 96% of all pixels, in which the percentage of pixels with CR between 0.05 and 0.2 reached 18.5%. Better crop growth was mainly distributed in the western and central Hubei province and the regions with the best growth concentrated on Xiangyang city. The crop growth was worse in the eastern Hubei province (such as the cities of Huanggang and Ezhou) compared with that in the past four years under similar meteorological conditions (see Figs. 1 and 2).

**Fig. 3.**
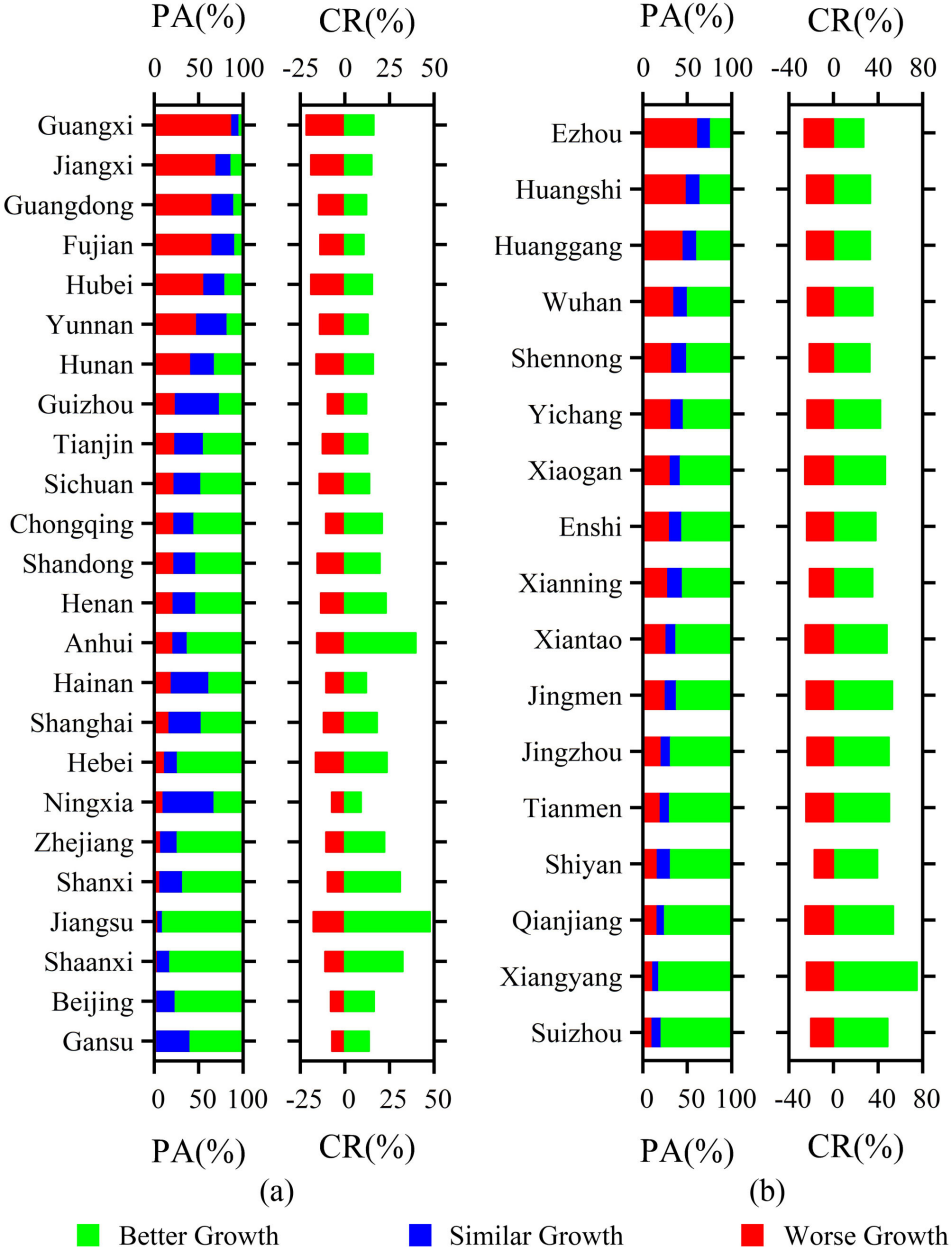
During the period of the rapid spread of COVID-19 in China, the PA for the crops with better growth, similar growth, and worse growth at (a) provincial scale in China and (b) city scale in Hubei province, respectively, as well as the relative CR for better growth and worse growth.

At the province scale, more than 70% of the crop in Hebei, Jiangsu, Zhejiang, and Shaanxi grew much better compared with that in the past four years under similar meteorological conditions. The relative CR was more than 20% and even 47% in Hebei province. In addition, there are 11 provinces where crops with better growth accounted for more than 50% and the relative CR of many of these provinces was more than 20% [see Fig. 3(a)]. The area of the crops with worse growth in provinces, such as Guangxi, Jiangxi, Guangdong, Fujian, Hubei, and Yunnan, accounted for 35% and the relative CR was about −12% [see Fig. 3(a)]. In Hubei province, the area of the crops with better growth in Xiangyang, Qianjiang, Tianmen, Shiyan, and Suizhou accounted for more than 70% and the relative CR was more than 30%. The area of the crops with worse growth in Ezhou and Huangshi city was over 40%. In Wuhan city, 35% of crops showed worse growth and the relative CR was about −23% [see Fig. 3(b)].

### B. Comparisons of Crop Growth Derived From Different Remote Sensing Data

**Fig. 4.**
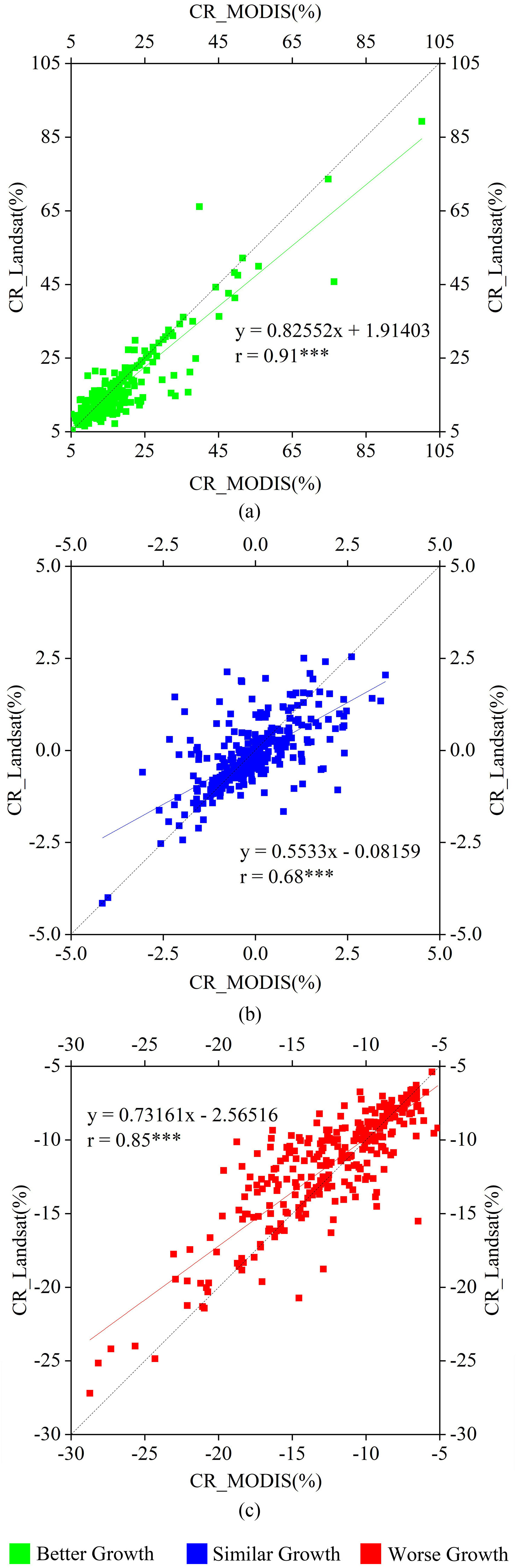
Comparisons of the relative CR of (a) better growth, (b) similar growth, and (c) worse growth based on 30 m LUCC product derived from Landsat and 463 m MCD12Q1 product at the city scale in China. *** indicates *p* < 0.001.

The primary remote sensing data involved in this study include: Sentinel-2 reflectance data with a spatial resolution of 10 m was resampled to 30 m; the LUCC product was generated by the 30 m Landsat series data; MOD09GA reflectance and MCD12Q1 land cover product with a spatial resolution of about 463 m. However, these two data still exist some uncertain. For example, agriculture is sometimes under-represented and labeled as the natural vegetation in some tropical areas in MCD12Q1 IGBP classification data because the cropland field sizes may be too small to be identified. Therefore, we investigated the consistency of the relative CRs derived from two different land cover products at the city scale (see Fig. 4). The significant correlations of the relative CRs were found between derived from 30 m LUCC product and 463 m MCD12Q1 product. The correlation coefficients of the relative CRs of crop growth with better growth, similar growth, and worse growth were 0.91, 0.68, and 0.85, respectively, and all these correlations were found to be very significant at the level of *p* < 0.001 (see Fig. 4). The significant linear relationship proved the consistency and reliability between MCD12Q1 IGBP land cover and LUCC. The main reasons include that the LUCC product was produced based on Landsat 8 data with good quality, and the producers have sufficient experiences and effective methods after six LUCC products in other years from 1980 to 2015, which are helpful for the LUCC product with high accuracy [51]–[53]. In addition, the areas were identified as the cropland in four years (from 2015 to 2018) were retained using the MCD12Q1 product, which also ensured the reliability of the cropland extraction in this study [62].

**Fig. 5.**
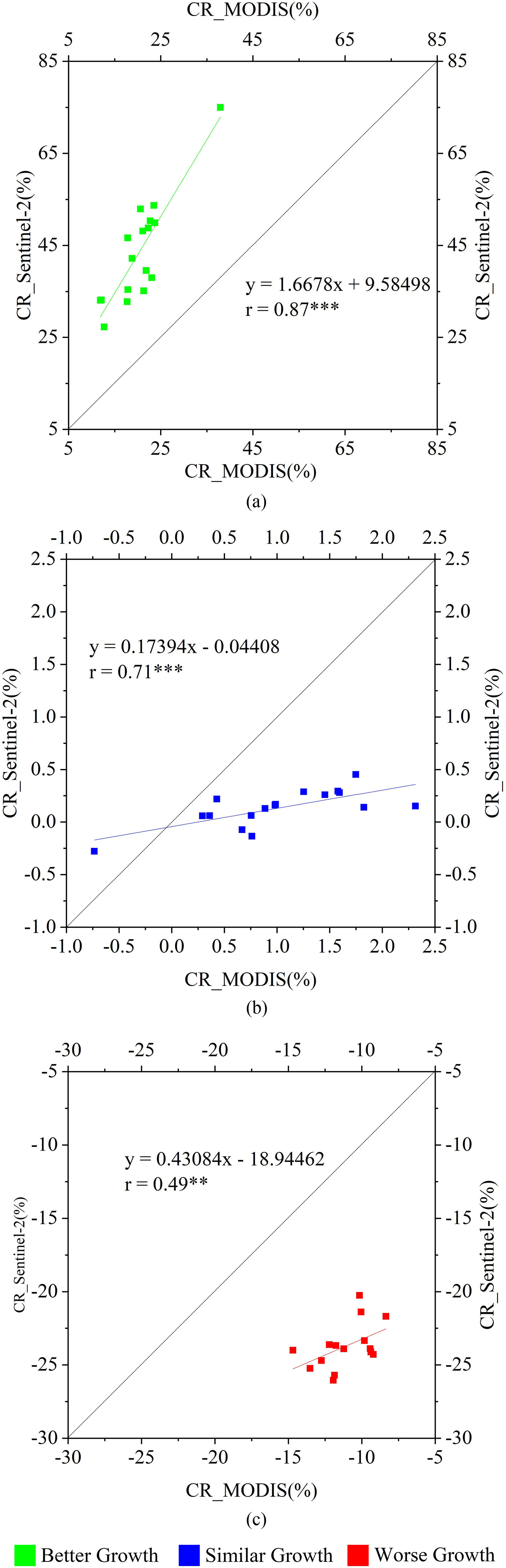
Comparisons of the relative CR of (a) better growth, (b) similar growth, and (c) worse growth derived from MOD09GA and Sentinel-2 EVI at the city scale in Hubei province. *** indicates *p*<0.001 and ** indicates *p*<0.05.

We also compared the relative CRs of crop growth based on 30 m Sentinel-2 EVI and 463 m MOD09GA EVI at the city scale in Hubei province. We intended to evaluate the influence of remote sensing data with different spatial resolutions on crop growth monitoring during the rapid spread period of COVID-19 in China. A general consistency of crop growth was observed between derived from 30 m Sentinel-2 EVI and 463 m MOD09GA EVI, especially for the relative CRs of crop growth with better growth and similar growth, and their correlation coefficients were 0.87 (*p* < 0.001) and 0.71 (*p* < 0.001), respectively. Except for Qianjiang and Shiyan province, the relative CRs of the worse growth of crops based on these two data also showed well relationship (*r* = 0.49 and *p* < 0.05). In addition, it was found that the relative CR calculated by Sentinel-2 EVI was generally larger than that by MOD09GA EVI for the relative CRs of the crop with better and worse growth (see Fig. 5). The well correlation ships proved the ability of these two to monitoring crop growth while scale effects might exist (such as smaller CR reflected by EVI from MODIS reflectance data).

### C. Relationship Between Crop Growth and Confirmed Cases of COVID-19 in China

**Fig. 6.**
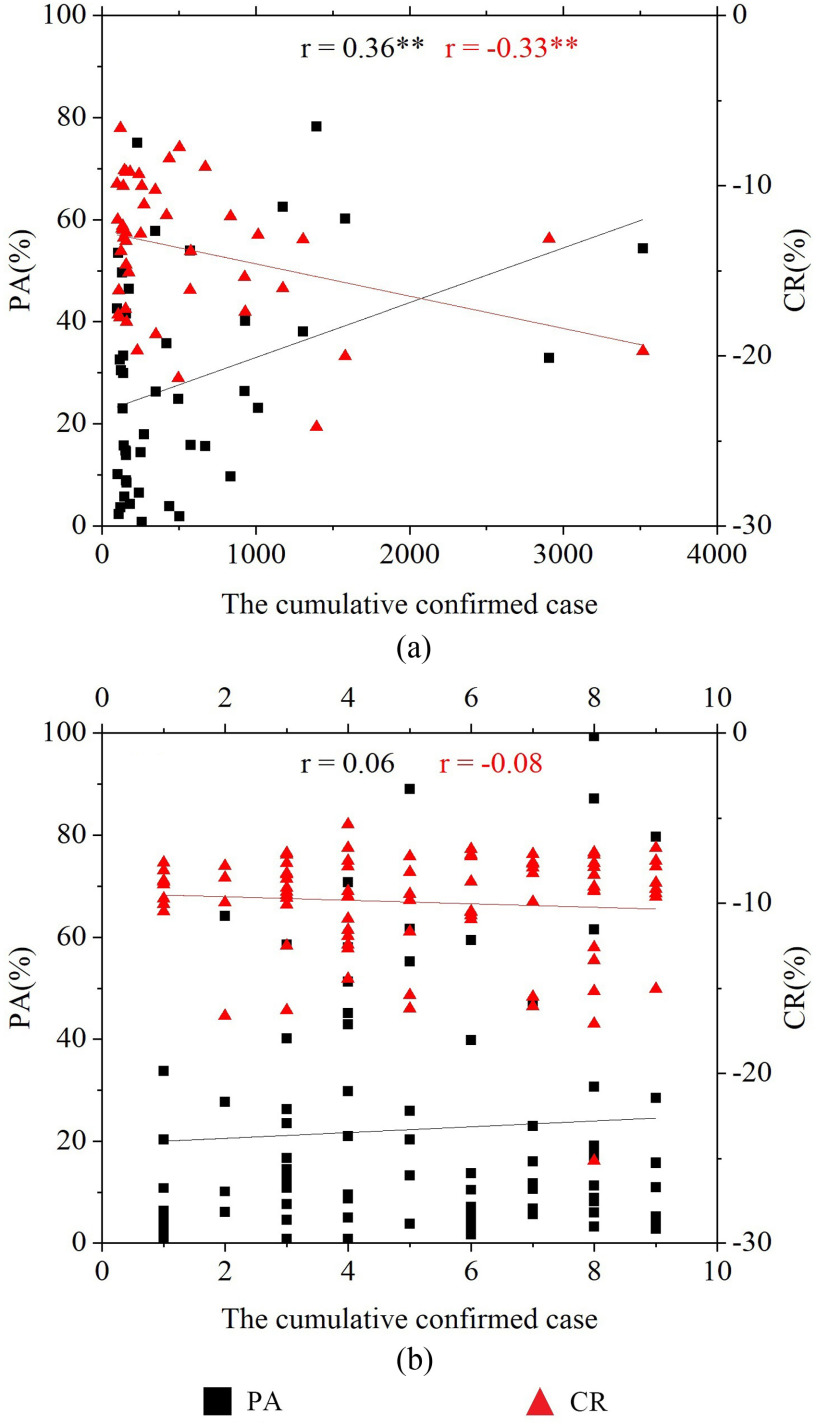
Relationships between the PA for the crops with worse growth (and also for the corresponding relative CR) and the cumulative number of the confirmed cases of COVID-19 at the city scale in China. (a) and (b) Results for the cumulative number of confirmed cases of COVID-19 are larger than 100 and less than 10, respectively. ** indicates *p* < 0.01.

The relationships between the PA for the crops with worse growth (and also for the corresponding relative CR) and the cumulative number of confirmed cases of COVID-19 at the city level in China were presented in Fig. 6. It was found that the PA for the crops with worse growth (and also for the corresponding relative CR) showed a significant correlation (*p* < 0.05) with cumulative confirmed cases when they were > 100 in the cities [see Fig. 6(a)] and such correlation was mainly attributed to several cities where cumulative confirmed cases > 500 according to the scatter distributions in Fig. 6(a). In addition, there was an insignificant relationship between crop growth and the cumulative confirmed cases in those cities with the cumulative confirmed cases less than ten [see Fig. 6(b)].

### D. Potential Causes for Crop Growth

In China, winter crops mainly include winter wheat, winter rape, and vegetables. The returning green stage of winter wheat is the key period for the final yield. During this period, the regrowth of winter wheat is mainly manifested in physiological processes, such as rooting, leaf spreading, and tillering. The field management measures, such as fertilization, pesticide spraying, and field watering, should be implemented during the returning green stage of winter wheat to ensure the healthy growth of winter wheat [63], [64]. In addition, the specific date of the returning green stage is mostly from mid-late February to mid-late March, which is mainly related to the locations of the winter wheat and the weather conditions [65]. For winter rape and winter vegetables, the growth would be affected by the weather conditions of chilling or continuous rainy. The field measurements, such as clearing the trenches, raising the borders to increase the temperature, and applying fertilizer timely, are necessary to reduce the damage to these crops [66]–[70]. The statistical data of historical weather forecast^1^^1^[Online]. Available: http://tianqi.2345.cn/. showed that some cities in Hubei province (e.g., Huanggang, Ezhou, and Wuhan) suffered from snow weather and continuous rain during the rapid spread period of the COVID-19 in China. Many field managements were needed but were challenging to be implemented in these regions. To prevent the spread of the COVID-19, measures, such as home isolation and traffic control, have been adopted. That might cause the reduction of necessary field management in some areas, especially in those cities with severe outbreaks, and indirectly affected crop growth, such as the PA for the crops with worse crop growth became larger when the cumulative confirmed cases increased in these cities with the cumulative confirmed cases > 500 [see Fig. 6(a)] and the corresponding CR in these cities was also larger than that in other cities. However, because COVID-19 pandemic outbreaks and spreads in the urban areas, few field managements are necessary during the period of the rapid spread of COVID-19 (i.e., from late January to midMarch 2020). The COVID-19 pandemic made no obvious impact on the overall growth of crops in China during the rapid spread period of the COVID-19 in China.

**Fig. 7.**
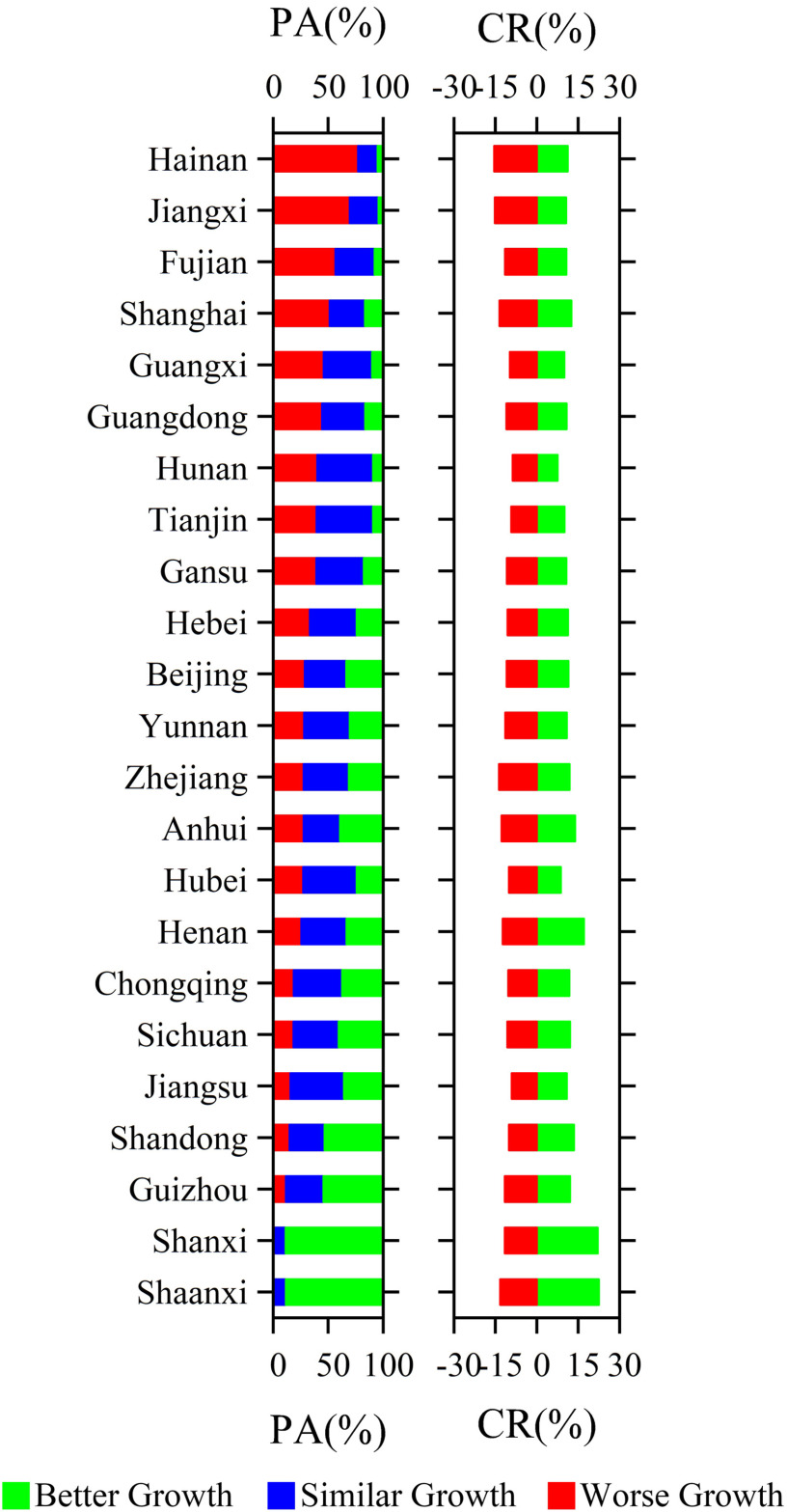
During the period of 12th March–30th June, the PA for the crops with better growth, similar growth, and worse growth at the provincial scale in China as well as the relative CR for better growth and worse growth.

It should be noted that crop growth is a continuous procedure, the emergence of an event does not only exert an influence during its happening time but also will have an effect on crops during the period after the event. Therefore, we investigated the EVI differences of corresponding areas during the period of March–May and counted the overall situation for each province. Before the statistic, we have extracted years with the most similar climate condition compared with the climate condition of 2020 during the period of March–May. In this way, we guarantee that crop growth in 2020 could be always compared with the crop growth in a similar year during these two periods (i.e., from 23rd January to 11th March and from 12th March to 31st May). The result is shown in Fig. 7. During the period from March to May, crops growth occurred in a significant variation. The area of crops with a similar growth increased and the area with worse growth was relatively stable. For many provinces displayed in Fig. 7, the proportion of crops with worse growth, similar growth, and better growth varied notably. For example, more crops with worse growth occurred in Hainan province and the area with worse crop growth decreased in Guangxi province. The crops in Shanxi and Shaanxi provinces showed more crops with better growth. Overall, the magnitude of CR of better and worse growth (14% and 12%, respectively) during the period from March to May became more balanced, while CR of better growth is predominant during the period from January to March. The area with better crop growth is the largest (about 43%), followed by the area of similar crop growth (about 35%), and finally, the area of crops with worse growth (about 22%). It is obvious that crop growth is a variable process and it is necessary to monitor crop growth in real time with the remote sensing technique to ensure food security [27], [29].

The main factors affecting the growth of crops include meteorological conditions and field management as well as other factors, such as the changes in the rotation system and seed improvement [20], [71], [72]. The monitoring crop growth in this study was based on similar meteorological conditions, which were derived from the daily temperature and precipitation of the ERA5 product. However, photosynthetically active radiation may also be the main factor affecting crop growth in some locations. Still, ERA5 products on GEE do not contain radiation data so far. For the crop with worse growth in southern China, such as the provinces of Guangdong and Guangxi, where the main crop is sugarcane and is in the spring sowing date during the period from late January to midMarch 2020. According to the historical weather data and field investigations [73], the worse growth of sugarcane during this period is mainly attributed to continuous rain and lack of light (i.e., photosynthetically active radiation). Similar meteorological conditions based on temperature and precipitation may also have some uncertainties in these locations. We weighted 0.3 for precipitation may also weaken the impact of precipitation on these areas. In addition, the crop growth during the period of rapid spread for COVID-19 in China in 2020 was compared with that during this same period in the past four years. Rotation system, seed improvement, and crop type might have some changes within the past five years and therefore might cause the discrepancy of EVI values but we did not isolate their influences on the crop growth due to the lack of these data. These might also bring some uncertainties for crop growth monitoring.

## IV. Conclusion

In this study, Sentinel-2 and MODIS images in the past five years were used to monitor the crop growth in China (especially Hubei Province) during the period of rapid spread for COVID-19 in China (i.e., from late January to mid-March 2020), and compared with the crops in the past four years under similar meteorological conditions. We also analyzed the impact of the severity of the COVID-19 pandemic on crop growth. In China, the winter crops were mainly distributed in the North China Plain and some regions in South China, the PA for the crops with better growth (51%) and the corresponding relative CR (17%) were significantly higher than the PA for the crops with worse growth (22%) and the corresponding relative CR (-14%), and the area of the crops with a similar growth accounted for 27%. In Hubei province, China, where the severest COVID-19 pandemic happened, the PA for the crops with worse growth in Ezhou and Huangshi was more than 40%, and that in Wuhan city was about 35%. At the city level, the significant level of correlation between the cumulative confirmed cases and the PA (and the corresponding CR) only reached 0.05 in those cities with the cumulative confirmed cases > 100, and this significant correlation was mainly attributed to the cities with the cumulative confirmed cases > 500. No significant correlations were observed in the cities in which the pandemic is not severe. We concluded that the COVID-19 pandemic has no obvious impact on the growth of winter crops during the rapid spread period of the COVID-19 in China and most of the crops in China showed a better growth status compared with that in the past four years.
